# Sensitive and Selective Voltammetric Sensor Based on Anionic Surfactant-Modified Screen-Printed Carbon for the Quantitative Analysis of an Anticancer Active Fused Azaisocytosine-Containing Congener

**DOI:** 10.3390/ijms24010564

**Published:** 2022-12-29

**Authors:** Jędrzej Kozak, Katarzyna Tyszczuk-Rotko, Krzysztof Sztanke, Małgorzata Sztanke

**Affiliations:** 1 Faculty of Chemistry, Institute of Chemical Sciences, Maria Curie-Skłodowska University in Lublin, 20-031 Lublin, Poland; 2 Laboratory of Bioorganic Compounds Synthesis and Analysis, Medical University of Lublin, 4A Chodźki Street, 20-093 Lublin, Poland; 3 Department of Medical Chemistry, Medical University of Lublin, 4A Chodźki Street, 20-093 Lublin, Poland

**Keywords:** fused azaisocytosine-containing congener with the *para*-nitrophenyl group, anticancer agent candidate, voltammetry, screen-printed sensor, anionic surfactant

## Abstract

3-(4-Nitrophenyl)-8-(2,3-dimethylphenyl)-7,8-dihydroimidazo[2,1-*c*][1,2,4]triazin-4(6*H*)-one (NDIT) is one of the most promising candidates for anticancer agents. Hence, a sensitive and selective sodium dodecyl sulfate-modified screen-printed carbon sensor (SPCE/SDS) was used for its quantitative analysis. The SPCE/SDS, in contrast to the SPCE, showed excellent behavior in the electrochemical reduction of NDIT by differential-pulse adsorptive stripping voltammetry (DPAdSV). Cyclic voltammetric (CV) studies reveal an irreversible, two-stage and not purely diffusion-controlled reduction process in 0.01 M HNO_3_. The sensor was characterized by CV and electrochemical impedance spectroscopy (EIS). Under the optimized conditions (t 45 s, ΔE 175 mV, ν 150 mV/s, and t_m_ 5 ms), the DPAdSV procedure with the SPCE/SDS presented a very wide linear range from 1 to 2000 nM and a low detection limit of 0.29 nM. A 1000-fold excess concentration of potential interferents commonly present in biological samples did not significantly alter the peak current of NDIT. The practical application of the proposed DPAdSV procedure with the SPCE/SDS was successfully checked by analyzing spiked human serum samples.

## 1. Introduction

**Figure 1:**
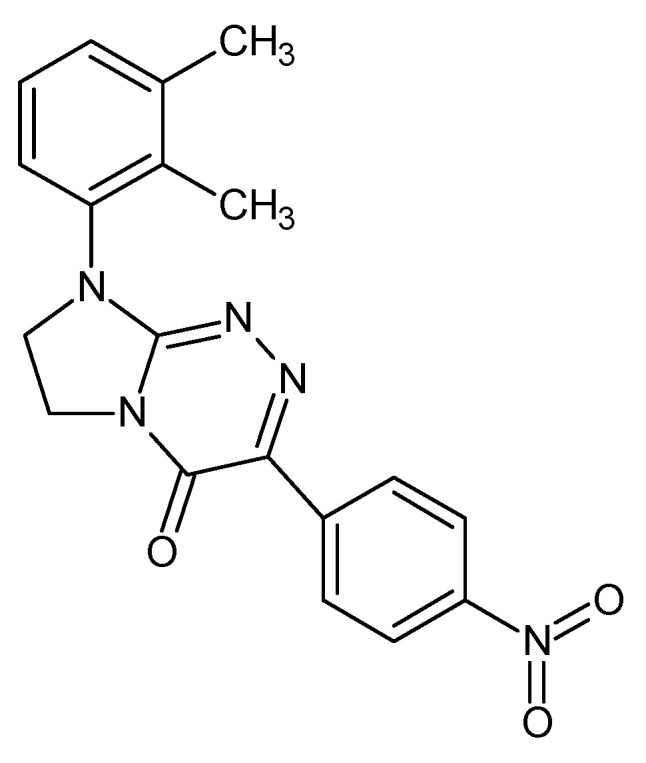
3-(4-Nitrophenyl)-8-(2,3-dimethylphenyl)-7,8-dihydroimidazo[2,1-*c*][1,2,4]triazin-4(6*H*)-one (NDIT)—an electroactive small molecule used in the present study.

Among antiproliferative active disubstituted fused azaisocytosine-containing congeners with fully defined and patented molecular structures [1,2], 3-(4-nitrophenyl)-8-(2,3-dimethylphenyl)-7,8-dihydroimidazo[2,1-*c*][1,2,4]triazin-4(6*H*)-one, abbreviated in this paper as NDIT (Figure 1), has previously been disclosed as one of the most promising candidates for anticancer agents [1]. This innovative nucleobase-like small molecule evoked a concentration-dependent growth-inhibitory effect in human epithelial tumor cells of the breast, cervix, lung, and ovary, which was the strongest in breast and cervical cancer cells [1,2]. In light of the current knowledge, the mechanism of its anticancer action may be related to the selective activation (via reduction with the participation of a number of flavoproteins) of this nitroaromatic-containing prodrug in tumor tissue into an anticancer active agent (i.e., the cytotoxic nitroanion radical and the cytotoxic hydroxylamine molecule) [1,3]. Furthermore, it has been found that NDIT—as a drug candidate for pharmaceutical use [2]—is distinctly less toxic towards normal epithelial Vero cells, reveals the optimal lipophilicity for high bioavailability after per os administration [1], as well as high thermal stability and high chemical purity [4]. These advantageous features indicate that this heterocyclic compound can be classified as a promising candidate for further in vivo pharmacological studies in the drug development process.

Taking into account the potential usefulness of NDIT in cancer chemotherapy [2], it is of great importance to develop a sensitive, selective, and reliable analytical procedure as the first method for its quantitative determination in solution, as well as biological samples. Such a method with optimized experimental and instrumental parameters could be used in the future to detect and determine the concentration of this substance in the blood serum of treated patients. It is supposed that such a method, after evaluating its linearity, selectivity, sensitivity, limit of detection, and limit of quantification, would have a chance for the prospective use in clinical analytics. Notwithstanding, as yet, no analytical procedure allowing the quantitative analysis of this small molecule has been developed to date or described. NDIT, as a drug candidate for pharmaceutical use, is the subject of our current electrochemical investigation due to the presence of an electroactive (prone to reduction both in in vitro and in vivo systems) *para*-nitrophenyl moiety at the *C*3 of the heterocyclic scaffold in its molecule. To date, no information about the electrochemical behavior of NDIT has been disclosed. Moreover, so far, no voltammetric sensor has been designed and applied for the quantification of NDIT.

In general, electrochemical methods are characterized by simplicity, the use of relatively inexpensive equipment, high sensitivity, speed, and high accuracy [5,6]. The main challenge of voltammetry as an electrochemical method is the fabrication of a suitable electrode. An ideal electrode should be mechanically stable and chemically non-reactive and should have a wide range of operating potentials [7]. Among the whole range of working electrodes used in voltammetry, in recent years, screen-printed electrodes (SPEs) are gaining more and more popularity. The screen-printing technique is considered to be an effective method of producing electrodes individually or in the form of entire electrode systems consisting of a working electrode, a reference electrode, and an auxiliary electrode. SPEs can be fabricated on different kinds of substrates, such as ceramic, glass, and flexible polymer polyimide, based on the characteristics of printing [8]. SPEs have been developed as single-use, disposable sensors for a variety of applications in environmental, clinical, and industrial analysis [9]. Disposable electrodes can be easily mass-produced, making them readily available and relatively inexpensive. Due to their small size, SPEs can be used with portable devices in field analysis. SPEs also allow working with small sample volumes. Moreover, these electrodes do not require laborious pretreatment and the cleaning of the surface. SPEs are easy to modify, which can be conducted by immobilizing the modifier on the electrode surface or by adding it to the ink that will be used to make the electrode [10,11,12,13,14,15].

Among the numerous modifiers of working electrodes used in voltammetry, we can distinguish surfactants. Due to their unique molecular structure, surfactants are widely used in the field of electrochemistry and electroanalytical chemistry for various purposes. They are often used as the selective masking agents to improve the selectivity and sensitivity of the electrochemical analysis [16]. The surfactant adsorbs on the electrode surface in the form of a layer that aggregates the electron allocation and enhances the peak current [17]. Furthermore, surfactants stabilize the electrochemical signal, enhancing the electron transfer rate and improving the detection limits [18]. Additionally, a medium containing a surfactant can prevent the fouling of the electrode [19,20]. There are many examples of voltammetric determinations with the use of surfactants in the literature. These are anionic surfactants such as sodium dodecyl sulfate (SDS) [15,20,21,22,23,24], cationic surfactants such as cetyltrimethylammonium bromide (CTAB) [18,25], and amphoteric [26] or non-ionic ones such as Triton X-100 [27].

Because NDIT may be useful in the future as a new anticancer agent, the present study was conducted with the goal of developing and optimizing the first analytical procedure allowing its quantitative determination based on a screen-printed carbon electrode (SPCE) modified with anionic surfactant, sodium dodecyl sulfate (SDS). The assimilation of SDS on the SPC surface of the electrode forms an adsorptive layer, which charges the surface negatively, prevents the accumulation of interferents, and enhances the NDIT peak current in acidic media. Therefore, an SDS-modified SPC sensor has outstanding electrochemical performance in highly sensitive and selective NDIT analysis. The results presented in this paper are important because the development of such a method, employing a reusable sensor, may be useful in the future for monitoring the concentrations of this potential anticancer agent in body fluids.

## 2. Results and Discussion

### 2.1. Characterization of SPCE and SPCE/SDS

**Figure 2:**
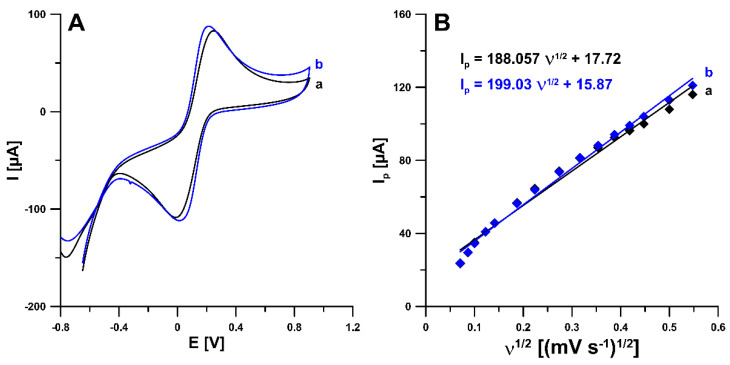
(**A**) Cyclic voltammograms of 5.0 mM K_3_[Fe(CN)_6_] and 0.1 M KCl at the SPCE (a) and SPCE/SDS (b) (ν of 150 mV/s). (**B**) The relationship between I_p_ and ν^1/2^ for ν from 5 to 300 mV/s.

The interfacial electron transport ability of the SPCE and the SDS-modified SPCE was analyzed using cyclic voltammetry (CV) and electrochemical impedance spectroscopy (EIS) in the K_3_[Fe(CN)_6_] solution, as well as in the HNO_3_ supporting electrolyte. Figure 2A shows CV curves at the SPCE and SPCE/SDS in 5.0 mM K_3_[Fe(CN)_6_] and 0.1 M KCl at a scan rate of 150 mV/s. According to the experimental results, the SPCE depicts a slightly lower Fe(II) and Fe(III) peak current intensity than the SPCE/SDS. This relationship translates into a similar active surface area (A_s_) of a modified electrode (0.0521 ± 0.0023 vs. 0.0551 ± 0.0028 cm^2^). It is worth adding that for A_s_ calculation, the relationship between the oxidation peak current of Fe and the square root of the scan rate (υ from 5 to 300 mV/s, Figure 2B), as well as the Randles–Sevcik equation, were used [28]. Moreover, the relative peak separations (χ^0^) were calculated for ν of 150 mV/s. It was found that the χ^0^ value of 2.86 for the SPCE/SDS is closer to the theoretical value (χ^0^ = 1) than the χ^0^ value of 4.12 obtained for the SPCE. In summary, the experimental results indicate that the SDS adsorption layer improves the electron transfer kinetics.

**Figure 3:**
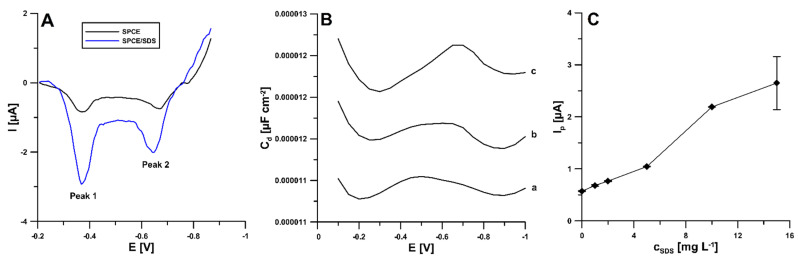
(**A**) Voltammograms of 50 nM NDIT obtained at the SPCE and SPCE/SDS (in the presence of 10 mg/L SDS). (**B**) The differential capacity–potential curves of the double-layer interface SPCE/supporting electrolyte in the presence of 0 (a), 10 (b), and 20 (c) mg L^−1^ SDS. (**C**) The relationship between the 50 nM NDIT peak current and SDS concentration from 0 to 15 mg/L.

The influence of the surfactant on the peak current of NDIT was analyzed by adding an increasing concentration of SDS to the supporting electrolyte. As shown in Figure 3A, the introduction of SDS (10 mg/L) allows for a significant increase in the 50 nM NDIT reduction signals (0.57 vs. 2.2 µA for peak 1 (peak potential of −0.37 V) and 0.54 vs. 1.4 µA for peak 2 (peak potential around −0.65 V)). In an acidic pH range, NDIT must exist in cationic form because the SDS molecules aggregate the species with alike charges and repel the species with like charges. SDS enhances the polarity on the exterior surface of the SPCE, as an outcome of which the enhancement of NDIT current signals can be observed. The adsorption of SDS on the SPCE surface was evidenced by the difference in the curves of the double-layer interface SPCE/HNO_3_ in the absence and presence of SDS (10 and 20 mg/L) (Figure 3B). The additional peak that appears at the potential of −0.7 V shows the desorption of previously adsorbed SDS during the recording of the differential capacity–potential curves. Moreover, Figure 3C shows the changes in the intensity of the 50 nM NDIT analytical signal with the changing concentration of SDS (0 to 15 mg/L). In the presence of 15 mg/L SDS, a large standard deviation of the peak current intensity was observed. Therefore, taking into account the NDIT peak current and the repeatability of the signal, an SDS concentration of 10 mg/L was selected for further measurements.

### 2.2. Electrochemical Reduction of NDIT over SPCE/SDS

**Figure 4:**
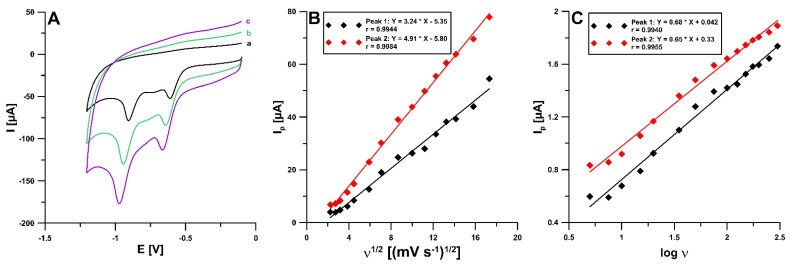
(**A**) Cyclic voltammograms of 0.01 M HNO_3_ at the SPCE/SDS for ν of 50 (a), 100 (b), and 150 (c) mV/s. (**B**) The relationship between I_p_ and ν^1/2^ for ν from 5 to 300 mV/s. (**C**) The relationship between I_p_ and log ν for ν from 5 to 300 mV/s.

**Figure 5:**
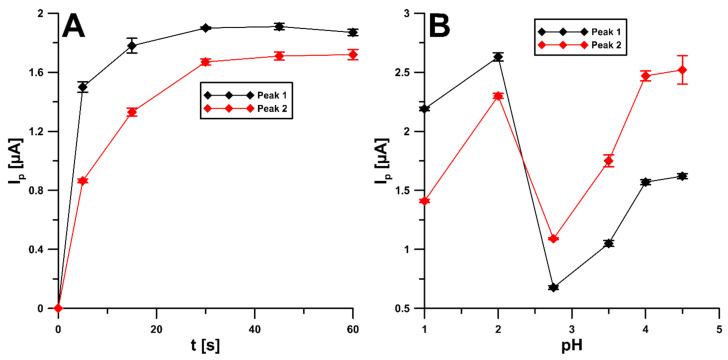
Influence of: (**A**) solution mixing time and (**B**) the type of supporting electrolytes (0.1 and 0.01 M solutions of HNO_3_, 0.1 M CH_3_COOH, and 0.1 M acetate buffers with a pH of 3.5, 4.0, and 4.5) on the 50 nM peak current of NDIT. The mean values of I_p_ are given with the standard deviation for n = 3.

The effects of the scan rate were evaluated in the range of 5–300 mV/s towards NDIT. Figure 4A shows the CVs at the SPCE/SDS with 10 mM NDIT for different scan rates (υ of 50, 100, and 150 mV/s). The electroreduction of NDIT occurs irreversibly and in two stages (potential peak 1 of −0.66 and peak 2 of −0.97 V for υ of 150 mV/s) in 0.01 M HNO_3_. The reduction signal responses have good linearity with the square root of the scan rates for the two NDIT peaks (Figure 4B). These results explained that the reduction of NDIT at the SPCE/SDS is followed by a diffusion-controlled process. However, the relationships between the log of the peak current and the log of the scan rate (Figure 4C) indicate that the process of NDIT reduction at the SPCE/SDS was not purely diffusion-controlled. Therefore, the influence of the selected potentials (0.1, 0, −0.1, and −0.25 V for 60 s) on the NDIT signal was examined. However, no improvement in the peak current was observed. Only examining the influence of mixing time (without applying the potential), before recording the signal, confirmed that the mixing solution would facilitate diffusion to the electrode surface and the adsorption of NDIT on the electrode surface (Figure 5A). Furthermore, the process of 50 nM NDIT reduction was studied in various supporting electrolytes (0.1 and 0.01 M solutions of HNO_3_, 0.1 M CH_3_COOH, and 0.1 M acetate buffers with a pH of 3.5, 4.0, and 4.5) (Figure 5B). The highest and best-shaped peak current with a low standard deviation was obtained for peak 1 in 0.01 M HNO_3_. Therefore, this solution was used in further studies.

**Figure 6:**
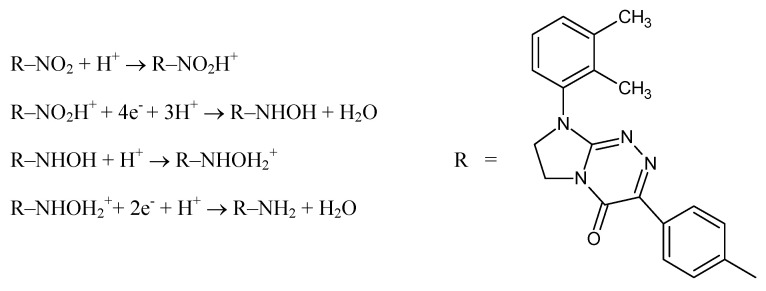
The hypothetical NDIT reduction mechanism.

As mentioned above, in the case of NDIT, an irreversible two-step reduction process took place at the surface of the SPCE/SDS as a sensor. The proposed electrochemical NDIT reduction course proceeding through an electron-gain mechanism is outlined in Figure 6. At the pretreatment step, due to the presence of strong nitric acid as the supporting electrolyte, the prior protonation [29,30] of NDIT to a cationic form at the sensor surface is most likely.

It has been proved in previous studies that the course of the electrochemical reduction of nitro-containing pharmaceutics strictly depends on the reaction conditions (e.g., the electrolyte solution pH and the electrode potential) [3,31,32]. Thus, the most probable course is that under acidic conditions the electrochemical reduction of NDIT in the first step leads to the NDIT hydroxylamine derivative, while in the second step (preceded by further protonation of the previously formed hydroxylamine derivative), it leads to the NDIT amino derivative, as shown in Figure 6.

### 2.3. Effect of DPV Parameters

**Figure 7:**
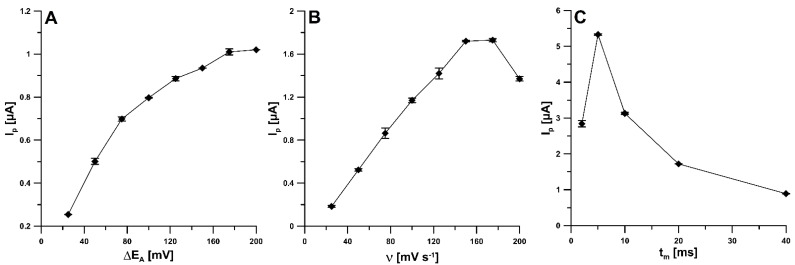
Influence of (**A**) ΔE_A_, (**B**) ν, and (**C**) t_m_ on the analytical signal of 50 nM NDIT. The mean values of I_p_ are given with the standard deviation for n = 3.

Further studies focused on the reduction peak 1 (the peak at less negative potential) due to the higher intensity of the peak current and the better repeatability of the signal. Much better reproducibility of the first signal (RSD of 3.5% for n = 10) was observed compared to the second signal (RSD of 10.7% for n = 10). Furthermore, the influence of the differential-pulse voltammetric (DPV) parameters (amplitude—ΔE_A_; scan rate—ν; and modulation time—t_m_) on the analytical signal of 50 nM NDIT was studied. In order to examine the influence of ΔE_A_ (from 25 to 200 mV), the NDIT reduction signal was measured (Figure 7A). The highest responses were obtained with an ΔE_A_ of 175 and 200 mV, so for further studies, the value of 175 mV was selected. Figure 7B represents the relationship between the ν in the range of 25–200 mV/s and the NDIT peak current. The highest responses were obtained with a ν of 150 and 175 mV/s, and therefore, a ν of 150 mV/s was selected as optimal. A t_m_ was studied in the range from 2 to 40 ms. As can be seen in Figure 7C, the highest NDIT signal was obtained for a t_m_ of 5 ms.

### 2.4. Sensitive and Selective Voltammetric Determination of NDIT

**Figure 8:**
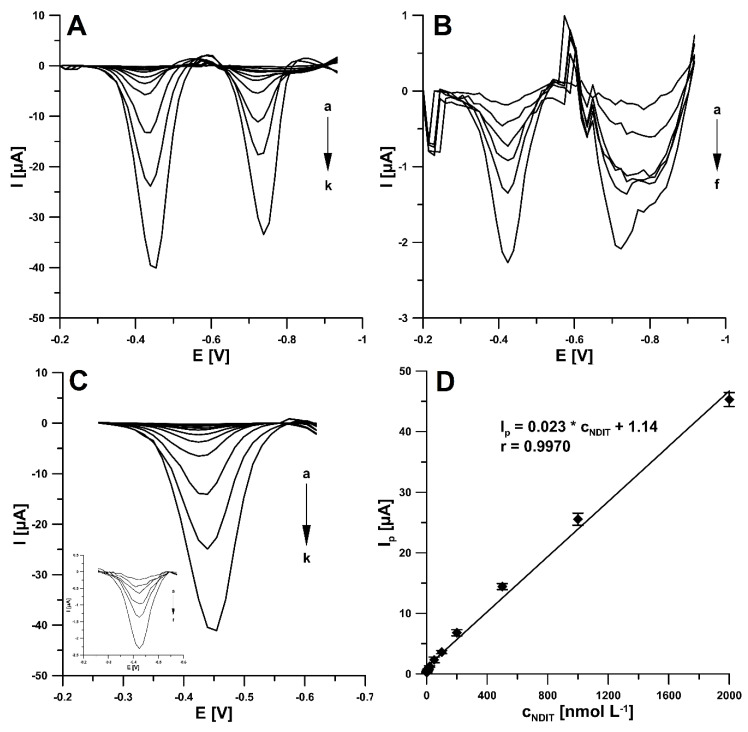
DPAdSV curves registered at the SPCE/SDS in 0.01 M solution of HNO_3_ containing 10 mg/L SDS and increasing concentrations of NDIT: (**A**) (a) 1, (b) 2, (c) 5, (d) 10, (e) 20, (f) 50, (g) 100, (h) 200, (i) 500, (j) 1000, and (k) 2000 nM; (**B**) (a) 1, (b) 2, (c) 5, (d) 10, (e) 20 and (f) 50 nM; (**C**) (a) 1, (b) 2, (c) 5, (d) 10, (e) 20, (f) 50, (g) 100, (h) 200, (i) 500, (j) 1000, and (k) 2000 nM; and insert in **C**) (a) 1, (b) 2, (c) 5, (d) 10, (e) 20, and (f) 50 nM. (**D**) Calibration plot of NDIT (peak 1). DPAdSV parameters: t of 45 s, ΔE of 175 mV, ν of 150 mV/s, and t_m_ of 5 ms. The mean values of I_p_ are given with the standard deviation for n = 3.

It is evident from Figure 8 that the NDIT reduction peaks at the SPCE/SDS increase with an increasing concentration of NDIT. The SDS film allowed better electron transfer on the SPCE surface and showed an extremely good linear response towards NDIT. As mentioned above, due to the higher intensity of the peak current (Figure 8A,B) and the better repeatability, the signal 1 is recommended for the quantitative analysis of NDIT. The reduction signal was found to be proportional in a very wide linear range of NDIT concentrations (1–2000 nM, Figure 8C,D). The detection limit (LOD) and quantification limit (LOQ) were calculated at 0.29 and 0.96 nM, respectively, using the following formulas: LOD = 3SD_a_/b and LOQ = 10SD_a_/b (SD_a_—standard deviation of intercept (n = 3); b—slope of calibration curve).

The interference studies were performed under the optimized conditions (0.01 M HNO_3_, 10 mg/L SDS and 50 nM NDIT) in the presence of various ions and organic substances commonly present in body fluids. On the basis of the conducted research, it was found that a 1000-fold excess of Fe(III), Ca(II), Mg(II), Cl(-I), glucose, epinephrine, ascorbic acid, and uric acid does not significantly alter the peak current of NDIT (does not cause changes greater than 10%). In the case of dopamine, a 100 times higher concentration than that of NDIT caused no significant influence on the NDIT response. In summary, the SPCE/SDS with a high interference withstanding ability permits the selective determination of NDIT.

### 2.5. Serum Sample Analysis

**Figure 9:**
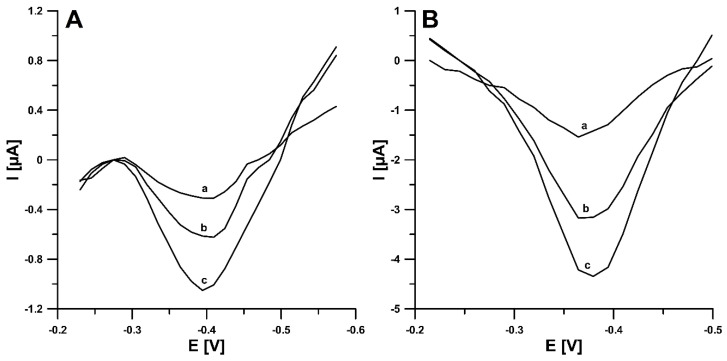
DPAdSV curves registered at the SPCE/SDS in 0.01 M solution of HNO_3_ containing 10 mg/L SDS during human serum analysis: (**A**) (a) sample + 20 nM NDIT, (b) as (a) + 20 nM NDIT, and (c) as (a) + 40 nM NDIT; (**B**) (a) sample + 50 nM NDIT, (b) as (a) + 100 nM NDIT, and (c) as (a) + 150 nM NDIT. DPAdSV parameters: t of 45 s, ΔE of 175 mV, ν of 150 mV/s, and t_m_ of 5 ms.

The DPAdSV procedure with the SPCE/SDS was used for NDIT determination in spiked human serum samples to prove its viability in real sample analysis. The samples were prepared as follows: 10 mL of human serum thawed at room temperature was spiked with 2.5 mL of 15% (*w/v*) TCA solution and the appropriate NDIT concentration. Then, the resultant solution was transferred to a centrifugal tube, centrifuged at 4000× *g* for 10 min, and filtered through a 0.22 µm Millipore filter. Next, 1 mL of sample was added to the supporting electrolyte, and the DPAdSV curves (Figure 9) were recorded under the optimized conditions. The recovery values were found to be very close to 100% (Table 1), which proves that the fabricated sensor is a reliable tool for the sensitive and selective determination of NDIT in real samples.

## 3. Materials and Methods

### 3.1. The Investigated Electroactive Molecule

For current electrochemical research needs, an electroactive NDIT molecule with a fully defined structure (i.e., 3-(4-nitrophenyl)-8-(2,3-dimethylphenyl)-7,8-dihydroimidazo[2,1-*c*][1,2,4]triazin-4(6*H*)-one [1,2], which is prone to reduction in biological systems, was chosen. The analyte was obtained from 1-(2,3-dimethylphenyl)-2-hydrazinylideneimidazolidine hydroiodide and ethyl 2-(4-nitrophenyl)-2-oxoacetate, according to the synthetic procedure, which was described in an earlier paper [1].

### 3.2. Instrumentation, Reagents, and Solutions

The electrochemical experiments were performed using a µAutolab analyzer (Eco Chemie, Utrecht, The Netherlands) controlled by GPES 4.9 software (CV, AdSV) or FRA 4.9 software (EIS) in a 10 mL quartz cell with a commercially available screen-printed sensor (Metrohm-DropSens, Oviedo, Spain). The sensors consisted of a carbon working electrode (diameter of 4 mm), a carbon auxiliary electrode, and a silver pseudo-reference electrode (SPCE, ref. 110).

The 1 M solution of HNO_3_, CH_3_COOH, and acetate buffers with a pH of 3.5, 4.0, and 4.5 were prepared from Merck (Darmstadt, Germany) reagents. Additionally, Merck standard solutions of Ca(II), Mg(II), Fe(III), and Cl(-I), as well as Sigma-Aldrich (Saint Louis, MO, USA) reagents (epinephrine, dopamine, uric acid, glucose, and ascorbic acid) were used in interference studies. The 1.0 and 0.1 mM solutions of NDIT were prepared in *N,N*-dimethylformamide (Sigma-Aldrich, Saint Louis, MO, USA). Normal human serum was purchased from Merck (Darmstadt, Germany). In order to precipitate proteins from serum samples, 15% (*w/v*) TCA solution (Sigma-Aldrich, Saint Louis, MO, USA) was used.

### 3.3. Fabrication of SPCE/SDS and Assay Procedure

The SPCE/SDS sensor was fabricated during the NDIT analysis. Under the optimized conditions, the supporting electrolyte consisted of 0.01 M HNO_3_ and 10 mg/L SDS. The 45 s solution mixing time before recording the signal facilitates the diffusion of the NDIT molecules from the solution to the electrode surface and the adsorption of the NDIT particles on the electrode surface. Sometimes, adsorption of SDS takes place on the surface of the SPCE. The DPAdSV curves were registered within the potential range from −0.2 to −1.1 V with amplitude–ΔE_A_–of 175 mV, scan rate–ν–of 150 mV/s and modulation time–t_m_–of 5 ms. The background curve was subtracted from each voltammogram and the baseline was corrected.

## 4. Conclusions

In summary, for the fabrication of the screen-printed carbon modified sodium dodecyl sulfate sensor (SPCE/SDS), we report the adsorption of anionic surfactant molecules on the electrode surface during the analysis. The unmodified SPCE and the SPCE/SDS sensors were characterized by cyclic voltammetry (CV) and electrochemical impedance spectroscopy (EIS). Furthermore, the SPCE/SDS sensor showed an electrochemical response towards 3-(4-nitrophenyl)-8-(2,3-dimethylphenyl)-7,8-dihydroimidazo[2,1-*c*][1,2,4]triazin-4(6*H*)-one (NDIT), one of the most promising candidates for anticancer agents. The substantial increase in the NDIT peak current with a sharper and well-defined peak at the SPCE/SDS reflects the faster electron transfer kinetics due to the presence of the SDS film. The SPCE/SDS sensor with its outstanding electrochemical performance is highly sensitive and selective for NDIT analysis. The DPAdSV procedure with the SPCE/SDS presented a very wide linear range from 1 to 2000 nM and a low detection limit of 0.29 nM. It should be highlighted that this is the first analytical procedure developed for the NDIT analysis. The results suggest a promising analytical tool for NDIT analysis in human serum samples.

## Figures and Tables

**Table 1:** Results of NDIT determination in human serum samples.

NDIT Concentration (nM) ± SD (n = 3)		
Added	Found DPAdSV	Recovery * (%)
20	20.1 ± 0.88	100.5
50	50.4 ± 1.44	100.8

* Recovery (%) = (Found DPAdSV × 100)/Added.

## Data Availability

The data presented in this study are available on request from the corresponding author.
